# Sexual dimorphism in jump kinematics and choreography in peacock spiders

**DOI:** 10.1242/jeb.249416

**Published:** 2025-02-12

**Authors:** Ajay Narendra, Anna Seibel, Fiorella Ramirez-Esquivel, Pranav Joshi, Donald James McLean, Luis Robledo-Ospina, Dinesh Rao

**Affiliations:** ^1^ School of Natural Sciences, Macquarie University, Sydney, NSW 2109, Australia; ^2^ School of Engineering and Information Technology, University of New South Wales, Canberra, ACT 2612, Australia; ^3^ Instituto de Biotecnología y Ecología Aplicada, Universidad Veracruzana, 91090 Xalapa, Veracruz, México

**Keywords:** Salticidae, Jumping spider, Locomotion, High-speed videography, µCT, *Maratus*

## Abstract

Jumping requires a rapid release of energy to propel an animal. Terrestrial animals achieve this by relying on the power generated by muscles, or by storing and rapidly releasing elastic energy. Jumping spiders are distinctive in using a combination of hydraulic pressure and muscular action to propel their jumps. Though males and females of jumping spiders vary in body mass, sex-specific differences in jumping have never been studied. Here, we investigated the sexual dimorphism in the jump choreography and kinematics of spiders. We used high-speed videography (5000 frames s^−1^) to record locomotory jumps of males and females of the Australian splendid peacock spider, *Maratus splendens*. Using micro-computed tomography (µCT) imaging, we identified the animals' centre of mass and tracked its displacement throughout the jump. Our study revealed that peacock spiders exhibited the fastest acceleration among all known jumping spiders. Males demonstrated significantly shorter take-off times and steeper jump take-off angles compared with females. Our findings suggest that the third pair of legs acts as the propulsive leg in both male and female spiders. As males of *M. splendens* use leg III as part of the courtship display, we discuss the extreme selection pressure on this leg that drives two significant functions.

## Teaser

**Summary:** High-speed videography reveals sexual dimorphism in the jump kinematics and choreography of the Australian splendid peacock spider, *Maratus splendens*, which exhibit the fastest acceleration among any known jumping spiders.

## INTRODUCTION

To execute a jump, animals require a quick burst of energy to launch themselves from a substrate, during which they coordinate various body parts to move synchronously within a brief period. Jumping is an energetically expensive form of locomotion (Burrows, 2006; Krasnov et al., 2004) that rapidly moves an animal either towards or away from a target. Many taxa have developed distinct strategies for jumping or leaping, including mammals, marsupials, amphibians and arthropods (Burrows et al., 2021; Delciellos and Vieira, 2009; Juarez et al., 2023; Kiørboe et al., 2010; Moore and Clifton, 2023). Animal jumps are classified as muscle-actuated or spring-actuated systems based on how they are powered. In arthropods, muscle-actuated jumps are seen in ants (Aibekova et al., 2023), lacewings (Burrows and Dorosenko, 2014) and bush crickets (Burrows and Morris, 2003), where animals rely on the power generated by the contraction of leg muscles (e.g. muscles in the coxae, trochanter and tibia). The volume of the muscle and its physiological properties limit the jump performance (Aibekova et al., 2023; Sutton et al., 2016). Spring-actuated systems, also referred to as catapult systems or power-amplification systems, as seen in grasshoppers, click beetles, true bugs and fleas, bypass muscular limitations and output more power by relying on internal structures to store energy, which is then rapidly released to trigger the jump (Bolmin et al., 2022; Patek et al., 2011).

Jumping spiders of the family Salticidae are exceptional jumpers, as their name suggests. They leap to cross gaps, navigate detours, capture prey and evade predators (Aguilar-Arguello et al., 2020; Bolmin et al., 2022; Brandt et al., 2021; Nabawy et al., 2018; Nelson, 2024; Patek et al., 2011). Salticids jump to targets at different elevations and often cross gaps 10–15 times their body length (Nelson, 2024). Spider jumps are neither muscle nor spring actuated; instead, they use an unusual strategy referred to as the semi-hydraulic system (Brandt et al., 2021; Nentwig et al., 2022; Parry and Brown, 1959a; Weihmann et al., 2012). Spider legs have flexor muscles for postural movements but lack the extensor muscles required to extend their legs and power jumps. The legs are extended by increasing the haemolymph pressure within them. This pressure is generated by the contraction of muscles in the cephalothorax, which pumps haemolymph into the leg. Currently, though jumps have been described in five genera of Salticidae – *Attulus*, *Habronattus*, *Phidippus*, *Portia* and *Trite* (Aguilar-Arguello et al., 2021; Brandt et al., 2021; Hill, 2006; Nabawy et al., 2018; Parry and Brown, 1959b) – sex-specific differences in jump kinematics have not been investigated. Male and female jumping spiders experience different selective pressures. Similar to many other spiders, females tend to have larger abdomens as they must accumulate resources for egg production. More uniquely, male salticids use leg waving as part of a courtship display and also to rapidly escape from females and male competitors. These differences are likely to affect locomotion in distinct ways in male and female spiders.

**Fig. 1.**
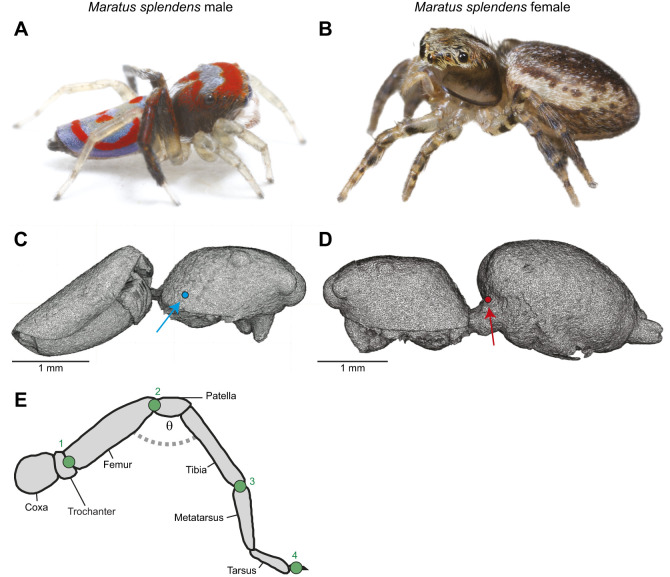
**The Australian splendid peacock spider, *Maratus splendens*.** (A,B) Colour photographs of a male and female spider. (C,D) 3D render from micro-computed tomography (µCT) scans of a male and female spider. The centre of mass (CoM) without legs is illustrated by a circle in a male (blue) and female (red) spider. (E) Schematic diagram of the spider's leg, illustrating the four joints that were tracked: (1) trochanto-femoral, (2) femoro-patellar, (3) tibio-metatarsal and (4) tarsal claw. We determined effective leg length (ELL) on every frame as the distance between the trochanter and tarsal claw divided by the sum of the length of each segment. In each frame, we also calculated the leg joint angle (θ). Photo credits: male, Ajay Narendra; female, Jürgen Otto.

Here, we investigated the jump kinematics and choreography of males and females of the Australian splendid peacock spider, *Maratus splendens* (Fig. 1A,B). Peacock spiders are native to Australia, with males renowned for their vibrant iridescent colours and elaborate courtship displays (Girard and Endler, 2014; Stavenga et al., 2016). During courtship, males of *Maratus* species spread open their brightly coloured opisthosomal flaps, which are usually folded around the abdomen, and oscillate around a female while waving the ornamented third pair of legs (Girard and Endler, 2014; Girard et al., 2021; Hill and Otto, 2011). Using high-speed videography, we studied locomotory jumps and determined the jump kinematics and choreography in male and female spiders.

## MATERIALS AND METHODS

We collected adult males and females of the Splendid Peacock spider, *Maratus splendens* (Rainbow, 1896) at the Lake Paramatta Reserve, Sydney, NSW, Australia. Spiders were individually housed in containers and provided with water and food (*Drosophila melanogaster*). Experiments were carried out between October and December 2023 at the Wallumattagal campus, Macquarie University.

### Experimental setup and filming

**Fig. 2.**
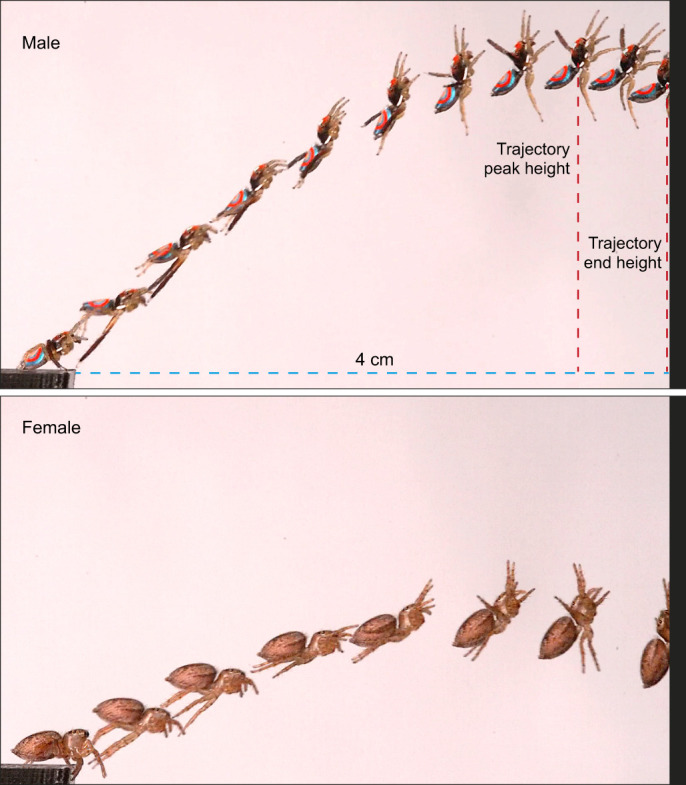
**Experimental setup and jump sequence in a male and female Australian splendid peacock spider, *M. splendens*.** Individual spiders jumped of their own accord from the take-off platform on the left to cross a 4 cm gap to reach a vertical landing platform. Spiders were filmed in profile at 5000 frames s^−1^. The time lapse series to illustrate the jump is shown for an example male (top) and female (bottom) spider. The images chosen are for clarity and are not spaced at regular time intervals. The two vertical dashed lines illustrate the trajectory peak height and trajectory end height.

Experiments were carried out in the laboratory with flicker-free LED lighting, and constant temperature conditions of 24°C. We fixed a landing and take-off platform to an optical bench which was placed on a vibration isolation table. The platforms were 3D printed with black PLA using Ultimaker S3. The take-off platform (4.0×0.5×0.1 cm, L×W×H) was horizontal to the ground on which a spider walked on their own from a collection jar. We placed a vertical landing platform (0.1×0.5×4.0 cm, L×W×H) 4 cm away from the take-off platform. Individual spiders jumped from the take-off to the landing platform of their own accord (Fig. 2). Thus, jumps were not elicited but allowed to happen naturally. We removed all other visual targets in the vicinity, which ensured that spiders carried out locomotory jumps to the landing platform. Most spiders jumped immediately, whereas some took a few minutes to jump towards the landing platform. For each individual, we recorded three consecutive jumps. The one exception was a female spider for which we recorded only two jumps, as this animal was reluctant to jump to the target on its third attempt. Animals were weighed immediately after they carried out the jumps (Sartorius Entris ii Advanced line Balance, Göttingen, Germany, 0.1 mg repeatability) and subsequently released back into their natural habitat.

We filmed the jumps with a 105 mm Sigma lens attached to a high-speed camera (T1340, Phantom, Adept Turnkey Pty Ltd, Osborne Park, WA, Australia) at 5000 frames s^−1^ (2048×1024 pixels). The spider and filming area were illuminated with Godox SL 300III light source. We filmed either the entire jump trajectory (male: *n*=4 spiders, 12 jumps; female: *n*=7 spiders, 20 jumps), or magnified views of take-off jumps to track leg joints accurately (male: *n*=6 spiders, 18 jumps; female: *n*=5 spiders, 15 jumps).

Raw footage was converted from cine to uncompressed mp4 files and trimmed in Videoloupe (Corduroy Code Inc., Vancouver, BC, Canada). Trimmed files were imported to DLTdv8 (Hedrick, 2008) in Matlab 2023b (Mathworks, Natick, MA, USA) where we carried out a manual frame-by-frame analysis to track different body structures and extract *x*,*y* coordinates. In both full-jumps and take-off-only jumps, we tracked the centre of mass (CoM; see below). From these coordinates, we reconstructed the jump trajectory. In full jumps, we identified the maximum height attained during a jump and the height above ground when animals reached the landing platform. We used the displacement of the CoM to determine jump kinematics, which included: (i) take-off duration (ms) – the duration between the first instance when acceleration increased from zero (i.e. the first frame where there was movement) to the first instance when all legs were off the ground. All kinematic measurements below were calculated for the same period as the take-off duration and maximum kinematic values for each jump was identified: (ii) take-off velocity (m s^−1^), *v*=*d*/*t*, where *d* is distance and *t* is time, determined for every frame; maximum take-off velocity was identified and used to calculate the following variables; (iii) acceleration (m s^−2^), *a=*Δ*v*/Δ*t*, where Δ*v* is change in velocity and Δ*t* is change in time, determined for every frame; peak acceleration was identified and used to calculate the following variables; (iv) kinetic energy (µJ), ke*=*½*mv^2^*, where *m* is body mass and *v* is velocity; jump force (mN), *F*=*m*×*a*, where *m* is body mass and *a* is acceleration; (v) jump power (mW), *P*=ke/take-off duration; (vi) ***g***-force, ***g****=a*/9.81; and (vii) take-off angle (deg): the angle of the CoM between the first frame prior to when the abdomen moves and the first frame when all legs are off the ground, relative to the plane of the substrate.

### Contribution of different legs to jumping

To identify the contribution of different legs in a jump, we tracked four leg joints in each leg in one randomly selected jump of all male (*n*=6) and female spiders (*n*=5) from the high-magnification footage. We tracked the following joints: trochanto-femoral, femoro-patellar, tibio-metatarsal and tarsal claw (Fig. 1E). Over the course of the jump, we determined leg extension using two methods, both adapted from Brandt et al. (2021). In the first, we calculated the effective leg length (ELL), which was the absolute distance between the trochanter and tarsal claw divided by the sum of length of all segments. Values range between 0 and 1, where values close to 1 indicate a fully extended leg. In the second, we determined the flex or the leg joint angle, i.e. the angle formed by the trochanto-femoral, femoro-patellar and patellar–tibial joints (Fig. 1E). Lastly, we identified the time difference between the moment the animals reached peak acceleration and the instant all their legs completely left the ground.

### Identifying the CoM

Specimens were fixed in 4% paraformaldehyde for 4 h and then rinsed in 0.1 mol l^−1^ phosphate buffer (3×10 min). Specimens were stained in 2% Lugol's solution for 48 h followed by washes in phosphate buffer (3×10 min). Samples were individually placed in small Eppendorf tubes filled with 70% ethanol for imaging for micro-computed tomography (µCT). We imaged the samples with a Bruker SkyScan1272 µCT scanner using a voltage of 45 kV, a current of 200 μA and a 0.5 mm aluminium filter. Projection images were reconstructed using SkyScan's NRecon v.2.2.0.6 software (Bruker, Kontich, Belgium) and data were output as a bitmap stack. Scans had a voxel resolution of between 1.5 and 5.0 µm.

The µCT image stacks were imported into Fiji (Schindelin et al., 2012). Scale information was added and a binning transformation (averaging) was applied to the *x*,*y*,*z* axes to halve the resolution in each axis to reduce the file size. Stacks were then exported as a NIfTI file. This file was opened in ITK-snap v.4.0.0 (Yushkevich et al., 2006) where we used the thresholding user guided auto-segmentation tool to segment the images. The resulting segmentation was manually refined to ensure there were no interior gaps and we assumed that the cephalothorax and abdomen were homogeneous and solid for CoM calculations. The legs were additionally separated into a different label as they were not included in the CoM calculation. Finally, a volume mesh was generated and exported as an STL file. The mesh was imported into MeshLab v.2022.02 (Cignoni et al., 2008), where the mesh was simplified using quadratic edge collapse decimation, isolated faces were removed and non-manifold edges were repaired before re-exporting as an STL file again. The cleaned mesh was loaded into AutoDesk Fusion 360 v.2.0.17721 where the CoM was identified.

### Statistical analyses

We used circular statistics, the Watson–Williams test in Oriana (Kovach Computing Services), to determine whether the mean take-off angles differed between male and female spiders.

We used linear mixed-effects models to examine the relationship between the sex of the spider, body mass and kinematic variables. The modelling was carried out in R using the ‘lme4’ and ‘lmerTest’ packages. The initial analyses involved fitting a full model with the sex of the spider and body mass as fixed effects. In addition, the model included an interaction term (body mass×sex) to evaluate the combined effect of these two factors. Individual spider IDs were used as random effects. The second model was similar but without the interaction term, assuming the relationship between the body mass and the kinematic variable does not differ by sex. The final model was determined based on Akaike's information criterion (AIC) by comparing the fits of the two models (Table S1). After selecting the optimal random effects' structure, the significance of the fixed effects was assessed using the *F*-test with Satterthwaite correction as implemented in the ‘lmerTest’ package.

## RESULTS

### Morphology

Males of *M. splendens* were distinctly lighter compared with their female counterparts (male: 4.94±1.86 mg, *n*=10; female: 10.40±2.05 mg, *n*=12; means±s.d.; Mann–Whitney, *U*=3.0, *P*<0.001; Table 1). The CoM in the male was located on the cephalothorax, just above the fourth coxae (Fig. 1C). The CoM in the female was located on the anterior-most region of the abdomen (Fig. 1D).

**Table 1.** Jump kinematics in Salticidae

Species (sex) *N* Gap size	Body mass (mg)	Acceleration (m s^−2^)	Take-off velocity (m s^−1^)	Take-off duration (ms)	Take-off angle (deg)	Kinetic energy (µJ)	Jump force (mN)	Jump power (mW)	***g***-force	Inter-frame interval (ms)
*Maratus splendens* (male) *N*=10 Gap: 4 cm	4.9	127.8	0.84	20.8	25.08	1.7	0.63	0.09	13.03	0.2
*Maratus splendens* (female) *N*=12 Gap: 4 cm	10.4	122.7	0.81	24.6	20.89	3.4	1.25	0.15	12.5	0.2
*Attulus pubescens* (male) *N*=2 Gap: 5 cm	10.0	51.3	0.67	13.06	12.0	2.24	0.51	0.17	5.23	11.9
*Habronattus conjunctus* (mixed) *N*=9 Gap: 4–5 cm	14.5	28.3	0.48	17.65	18.7	1.76	0.40	0.10	3.18	1.0
*Phidippus princeps* (female) *N*=1 Gap: 6 cm	150.0	51.4	0.83	16.15	22.0	51.6	7.71	3.20	5.2	15.0
*Phidippus regius* (female) *N*=1 Gap: 3–6 cm	150.0	36.07	0.79	22.19	19.67	48.36	5.41	2.24	3.68	0.31

Values shown are average values for each criterion. For *Habronattus conjunctus*, we used data with jump gaps of 4 cm and 5 cm (Brandt et al., 2021). For *Phidippus regius*, we used data with jump gaps of 3, 4.5 and 6 cm, which were in the same horizontal plane (Nabawy et al., 2018). Data for *Attulus pubescens* were derived from Parry and Brown (1959b) and those for *Phidippus princeps* were from Hill (2006).

### Overview of jump kinematics

**Fig. 3.**
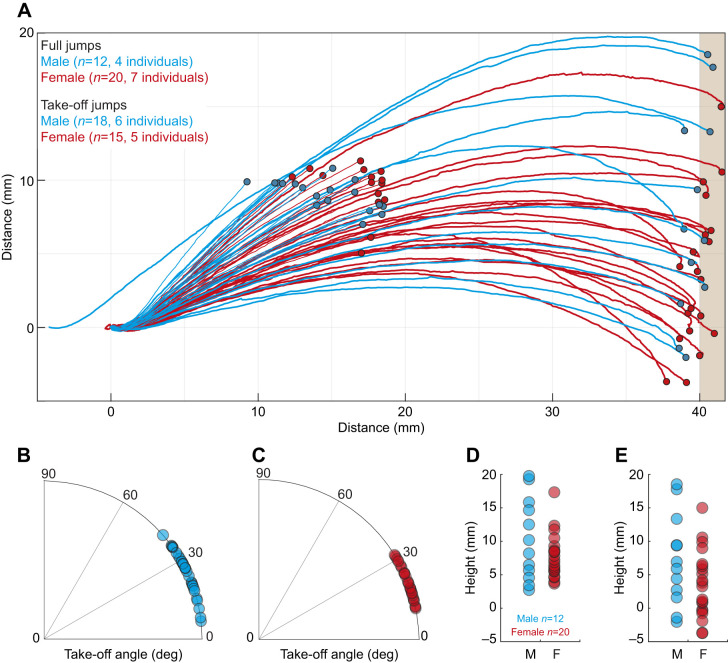
**Jump trajectories and characteristics in male and female Australian splendid peacock spiders, *M. splendens*.** (A) Jump trajectories start at 0,0, except for that of one eager male, which jumped before reaching the edge of the take-off platform. Spiders jumped to a vertical target placed 40 mm away. Trajectories shown are for full jumps: complete trajectories from take-off to landing, and take-off jumps: trajectories from take-off to up to 15 cm from the origin. The CoM was tracked in every frame to construct the trajectory. The endpoint of each trajectory is shown by a small circle. (B,C) Distribution of take-off angles in males (blue; B) and females (red; C). Each circle represents the take-off angle of one jump. (D,E) Trajectory height at the peak (D) and end (E) of the jump. For D and E, we only used data from full trajectories.

We studied the jump kinematics of 10 male (30 jumps) and 12 female spiders (35 jumps) and on every occasion, all animals jumped across the 4 cm gap to successfully reach the landing platform (Fig. 3).

The smallest individual filmed was a 2.0 mg male that had a take-off duration of 22 ms, peak acceleration of 141.80 m s^−2^ and maximum take-off velocity of 0.89 m s^−1^. In comparison, the heaviest individual filmed was a 13.2 mg female that had a take-off duration of 14 ms, peak acceleration of 122.33 m s^−2^ and a maximum take-off velocity of 0.76 m s^−1^. Between the two sexes, the highest and lowest acceleration was seen in female spiders (155.22 and 90.06 m s^−2^). Between the sexes, the highest take-off velocity was in males (0.98 m s^−1^) and the lowest was in females (0.61 m s^−1^). There was a noticeable overlap in body mass between the sexes, where the heaviest males were heavier than the lightest females.

### Effects of body mass and sex on jump kinematics

**Fig. 4.**
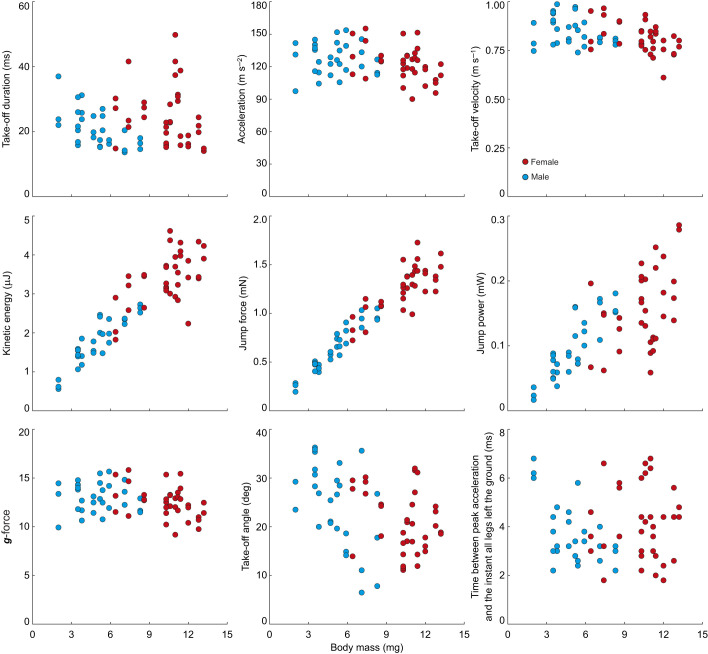
**Relationship between jump kinematics and body mass in male and female Australian splendid peacock spiders, *M. splendens*.** Scatter plots show the relationship of body mass with take-off duration, peak acceleration, maximum take-off velocity, kinetic energy, jump force, jump power, ***g***-force, take-off angle and the time between the moment the animals reached peak acceleration and the instant all their legs completely left the ground.

Body mass had a significant effect on all the kinematic variables we measured (Fig. 4, Table 2). Sex of the spider had a significant effect on all kinematic variables except take-off velocity (Fig. 4, Table 2). After controlling for body mass, we found that all kinematic variables, with the exception of jump power, were lower in males compared with females (Table 2). We found a significant interaction between sex and body mass for all kinematic variables except take-off velocity and kinetic energy (Fig. 4, Table 2).

**Table 2.** Statistical outputs of the linear mixed-effects model

Parameters	Fixed effects						Type III ANOVA			
		Estimate	s.e.	d.f.	*t*-value	*P*-value	Num d.f.	Den d.f.	*F*-value	*P*-value
Take-off velocity	Intercept	−0.032	0.083	62	−0.391	n.s.				
	Mass	−0.077	0.035	62	−2.172	<0.01	1	62	4.716	<0.05
	Sex (male)	−0.016	0.035	62	−0.46	n.s.	1	62	0.211	n.s.
Acceleration	Intercept	5.347	0.227	61	23.525	<0.001				
	Mass	−0.023	0.098	61	−2.409	<0.05	1	61	4.073	<0.05
	Sex (male)	−0.515	0.244	61	−2.111	<0.05	1	61	4.456	<0.05
	Mass:Sex	0.243	0.112	61	2.161	<0.05	1	61	4.668	<0.05
Kinetic energy	Intercept	−0.203	0.323	61	−0.629	n.s.				
	Mass	0.607	0.139	61	4.375	<0.001	1	61	91.785	<0.001
	Sex (male)	−0.708	0.346	61	−2.044	<0.05	1	61	4.18	<0.05
	Mass:Sex	0.318	0.16	61	1.991	n.s.	1	61	3.962	n.s.
Jump force	Intercept	−1.561	0.0227	61	−6.866	<0.001				
	Mass	0.765	0.098	61	7.828	<0.001	1	61	247.772	<0.001
	Sex (male)	−0.514	0.244	61	−2.111	<0.05	1	61	4.456	<0.05
	Mass:Sex	0.243	0.112	61	2.161	<0.05	1	61	4.669	<0.05
Jump power	Intercept	−4.767	0.392	42.538	−12.145	<0.001				
	Mass	1.217	0.164	45.871	7.405	<0.001	1	45.872	54.828	<0.001
	Sex (male)	0.377	0.165	43.164	2.283	<0.05	1	43.164	5.213	<0.05
Take-off duration	Intercept	3.923	0.321	52.713	12.223	<0.001				
	Mass	−0.335	0.133	56.123	−2.513	<0.05	1	56.123	6.314	<0.05
	Sex (male)	−0.376	0.135	54.012	−2.785	<0.01	1	54.012	7.754	<0.01
***g***-force	Intercept	3.063	0.227	61	13.479	<0.001				
	Mass	−0.235	0.097	61	−2.409	<0.05	1	61	4.073	<0.05
	Sex (male)	−0.514	0.243	61	−2.111	<0.05	1	61	4.456	<0.05
	Mass:Sex	0.243	0.112	61	2.161	<0.05	1	61	4.668	<0.05

n.s., not significant (*P*>0.05).

Males had a significantly higher take-off angle compared with females (males: 25.08±8.55 deg, *r*=0.98, *n*=30; females: 20.89±6.25 deg, *r*=0.99, *n*=35; means±s.d.; length of mean vector; Watson–William test *F*=5.011, *P*=0.029; Figs 3B,C, 4, Table 1). From full jump trajectories, we measured the maximum height of the trajectory and trajectory height when spiders reached the landing platform (Fig. 2) and found no differences between sexes (Mann–Whitney: maximum trajectory height: *U*=98.0, *P*=0.408; trajectory end height: *U*=98.0, *P*=0.146; Fig. 3D,E).

### Leg kinematics

**Fig. 5.**
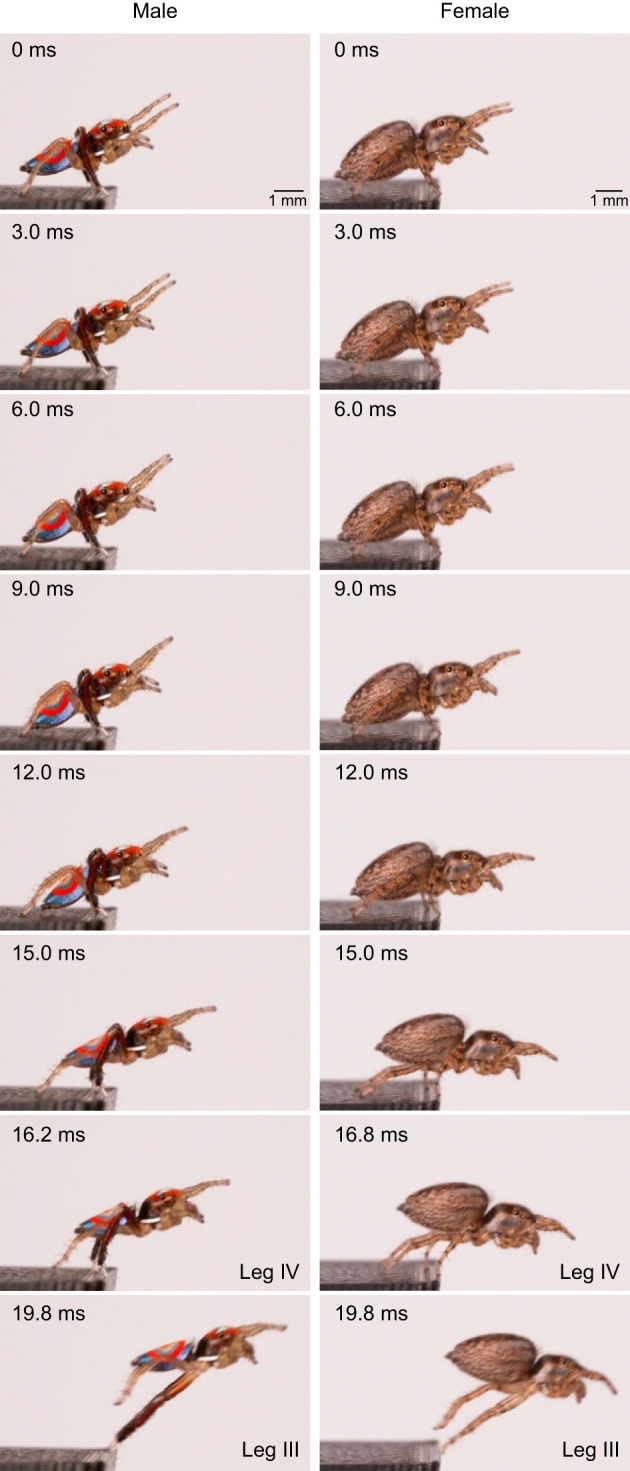
**An example of a jump sequence in a male and female Australian splendid peacock spider, *M. splendens*.** Images are shown of the following time frames: 0 ms: pose held in the last frame before movement where legs I and II were raised and extended; 3–9 ms: abdomen tip gradually moves down to touch the ground; 12 ms: leg IV begins to extend and CoM position moves forward; 15 ms: leg IV is mostly extended, leg III joint is acute, CoM position raises up; 16.2 ms/16.8 ms (male/female): first instance when legs IV left the platform; 19.8 ms: legs III left the platform.

The sequence of leg and body movements between the male and female spiders was similar. Prior to jumping, spiders of both sexes raised and extended their first two pairs of legs (Figs 2 and 5; Movie 1). Spiders waved their first two pairs of legs for 0.4–60 ms, and gradually lowered their abdomen. The tip of the abdomen touched the take-off platform briefly (∼0.2 ms; equivalent to our interframe interval), presumably to attach a drag line thread. The fourth pair of legs left the take-off platform first, followed by the third pair (Fig. 5; Movie 1). In females, the third pair of legs left the platform 3.5±0.4 ms (means±s.d., *n*=35) after the fourth pair, whereas in males it was slightly delayed (3.8±1.8 ms, *n*=30; Mann–Whitney *U*=216, *P*<0.001; Fig. 5; Movie 1).

We identified the duration between the moment the animals reached peak acceleration and the instant all their legs completely left the take-off platform. In both sexes, the fourth pair of legs left the platform close to when peak acceleration occurred (females: 0.4±0.2 ms after peak acceleration, means±s.d., *n*=35; males: 0.3±0.6 ms before peak acceleration, *n*=30). This was true for almost all jumps except one jump in a male (out of 30) and a female (out of 35). In both sexes, the third pair of legs left the platform after peak acceleration (Fig. 5; Movie 1; males: 3.4±1.4 ms after peak acceleration, *n*=30; females: 4.0±1.5 ms after peak acceleration, *n*=35).

**Fig. 6.**
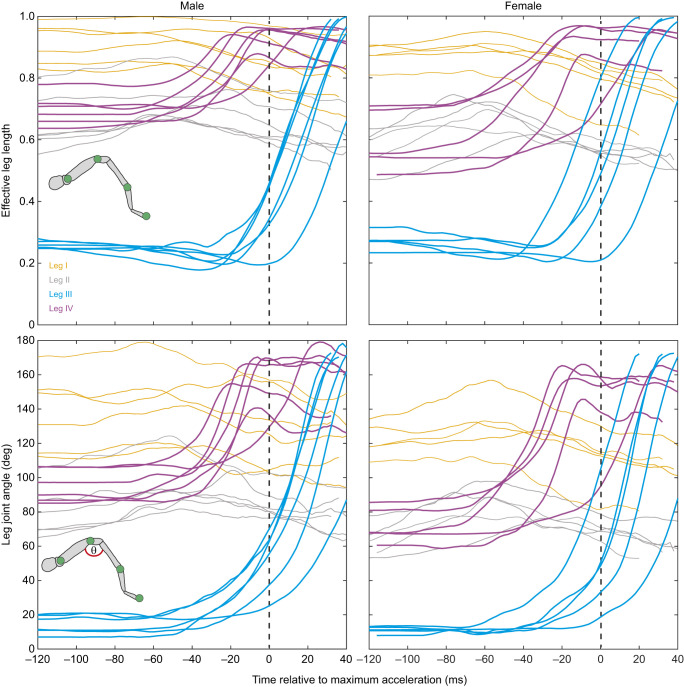
**Choreography of leg movements relative to peak acceleration during a jump in male and female Australian splendid peacock spiders, *M. splendens*.** Joints of four legs were tracked in 6 male and 5 female spiders. Each colour represents a leg. Inset shows a schematic diagram of a spider leg with the tracked leg joints (green circles) and the leg joint angle (θ). Top row: effective leg length is the fraction of the absolute distance between the trochanter and tarsal claw divided by the sum of the length of all segments. Values close to 1 indicate the leg is fully extended. Bottom row: the flex in the joint was determined as the angle formed by the trochanto-femoral, femoro-patellar and patellar–tibial joints. The leg movements were shifted to ensure peak acceleration was at 0 ms (vertical dashed line).

In a smaller sample size (6 males, 5 females, 1 jump each), we determined the extension and the timing of leg movement by calculating the ELL and angle of the leg joint relative to acceleration (Figs. 1E and 6). The ELL of leg IV in males and females gradually increased and was fully extended before animals reached peak acceleration (with one exception in a male and a female; Figs 1E and 6). In both sexes, maximum ELL and the least acute angle in leg III occurred after peak acceleration (Fig. 6).

## DISCUSSION

Male jumping spiders must execute rapid jumps to escape attacks from female and male competitors during courtship and to avoid being preyed upon. Hence, we focused on characterising sex-specific differences in the kinematics and choreography of jumps in the Australian splendid peacock spider *M. splendens*. Though we studied only mature adults, the body mass varied between individuals within both males and females. The variation in body mass in males (2.0–8.3 mg) and females (6.4–13.2 mg) could result from slightly different feeding habits, as well as the additional energy reserves females require for egg production. On average, females weighed more than twice as much as males, and the heaviest female was 6.6 times heavier than the lightest male.

The acceleration of the peacock spider, *M. splendens*, was the fastest among all known jumping spiders: more than twice as fast as the previous highest acceleration in *Phidippus princeps* and more than 4 times faster than that of *Habronattus conjunctus*, which had the slowest acceleration among all Salticids (Table 2; Brandt et al., 2021; Hill, 2006). The fast acceleration in peacock spiders may be an indication of the predation pressure or the speed of the prey they need to track and capture in natural environments. Although the lighter males had higher acceleration compared with the heavier females, after controlling for body mass, we found that acceleration in males was slower compared with that in females (Fig. 4, Table 2).

The lighter males had significantly shorter take-off times compared with those of females (Table 1). On average, males and females experienced the equivalent of 13.03 ***g***-force and 12.5 ***g***-force, respectively, during take-off, which is higher than any other known jumping spider (Table 1). In comparison, froghoppers (*Philaenus* sp.) that use stored energy for their jump, experience 550 ***g***-force (Burrows, 2006). As the ***g***-force experienced by *M. splendens* and other Salticids is several magnitudes lower than that of animals that rely on stored energy, the semi-hydraulic system that drives jumping in spiders is unlikely to have any elastic or stored energy component. Although the lighter males had a steeper jump take-off angle compared with the females (Fig. 3B,C), there was a large spread in both sexes for this measure. Surprisingly, the difference in the take-off angle between sexes was not reflected in differences in the maximum height of the jump trajectory (Fig. 3A,D,E). A potential reason for steeper take-off angles in males compared with females is their small body mass and reduced muscle power output. Male spiders, being lighter than females, take off at steeper angles to achieve the same height with less energy. Females, being heavier, may not need as steep an angle to reach the same height because of their heavier body, which provides more inertia. Because the target, the landing platform, was at the same distance for all spiders, males and females appear to have optimised their take-off angle to match their differing morphologies to achieve similar trajectory heights. Sexual dimorphism in locomotory strategies is known in spiders and insects. In spiders, small-bodied males outperformed heavier females in climbing vertical features or walking faster (Moya-Laraño et al., 2002; Prenter et al., 2010). In the oriental fruit moth, *Cydia molesta*, females outperformed males in velocity and flight distance (Hughes and Dorn, 2002), and in the butterfly *Euphydryas phaseton*, female flights are sluggish compared with those of their male counterparts, which is likely to be due to their greater wing loading, which requires a higher wing-beat frequency (Gilchrist, 1990). In ants, males lead a life on the wing, female workers are exclusively pedestrian, and reproductive females initially lead a life on the wing and subsequently become pedestrian (Narendra et al., 2011). While sexual dimorphism is prevalent in jumping spiders including *M. splendens*, they have been studied mostly in the context of courtship displays (Girard and Endler, 2014; Hill and Otto, 2011), and investigation into their foraging habits, spatial memory and biomechanics is lacking.

### Choreography of leg movements

Peacock spiders are best known for the elaborate courtship display that males carry out to entice the female (Girard et al., 2011, 2021; Hill and Otto, 2011). As part of this ritual, males of *M. splendens* extend, wave and lower the third pair of legs in front of the female. In contrast to the other legs, the third pair tend to be long and dark with bright white tarsal setae, indicating leg III is under selection pressure. While the third pair of legs are considered long, measurements of the leg have not been reported. Hence, we measured the length and 2D area of the leg segments by dissecting legs III and IV in one male and one female of *M. splendens*. Leg III was 1.23 times longer in the male than in the female; leg IV was 1.04 times longer in the male than in the female. In the male, leg III was the longest, with the femur being the longest segment. In the female, leg IV was the longest, with the metatarsus+tarsus section being the longest segment. The 2D area of the femur was largest in the male (leg III: 0.386 mm^2^; leg IV: 0.348 mm^2^) versus the female (leg III: 0.303 mm^2^; leg IV: 0.308 mm^2^). In comparison to female spiders, male spiders invested more in their femurs, which are larger in leg III than in leg IV, at least in two-dimensional space.

The third and fourth pair of legs played a crucial role in the jumps of both males and females. Well before the start of the jump or displacement of the CoM, in both sexes, spiders raised the first two pairs of legs and extended them in front of their cephalothorax (Fig. 5; Movie 1). The fourth pair of legs left the platform next, followed a few milliseconds later by the third pair. In most jumping spiders, similar to *M. splendens*, the third pair of legs are the last to leave the ground. The only exception is *Attulus pubescens*, in which the fourth pair left the ground last (Parry and Brown, 1959b). We followed the elegant strategy that Brandt et al. (2021) developed to identify the propulsive leg, by calculating the ELL and the angle between leg joints relative to peak acceleration. It was evident that spiders extended and held the first and second pair of legs straight, well before peak acceleration occurred. Leg IV attained the highest ELL and its leg joint was almost straight before the animals attained peak acceleration, with one exception, in all male and female spiders (Fig. 6). This meant that spiders continued to accelerate after leg IV was fully extended and had left the ground. Leg III reached the highest ELL after peak acceleration had occurred. These two findings suggest that leg III is the propulsive leg in both males and females of *M. splendens*, similar to findings in *H. conjunctus* (Brandt et al., 2021). Thus, leg III is under extreme selection pressure as it drives two significant functions: locomotory jumps and courtship behaviours. It remains to be seen whether males are more vulnerable to predation during courtship, as leg III is extended and waved as part of a visual signal. Equally, it is essential to characterise the 3D volume of the femur and investigate the internal structure and muscles involved in the jumps.

### Jump context

Jumping spiders occupy 3D-cluttered environments where they jump under various contexts. Here, we studied jumps exclusively in the context of locomotion that was planned and well executed. Escape or startle jumps provide a strikingly different context from locomotory jumps, with the former usually unsteady, non-directed and tending to have high accelerations. Identifying the kinematics of escape or startle jumps will pinpoint the limits of the system, which locomotory jumps do not capture (Kiørboe et al., 2010). Thus, the currently known acceleration and take-off velocity in *M. splendens* and *H. conjunctus* are likely to be lower than the maximum kinematics these species can achieve (Brandt et al., 2021). A second and unusual context is multiple jumping or continuous jumping, which is a form of endurance locomotion that jumping spiders and some insects exhibit (Ali et al., 1992). Here, we studied only a single jump, and it remains unknown how acceleration and jump forces change when animals perform consecutive jumps. A third context is the plane of jumping, i.e. whether an animal carries out horizontal, ascending or descending jumps at different elevations. Here, we studied jumps towards a 4 cm vertical target where animals could land anywhere on the target. Jumping to smaller targets at higher planes will probably require more energy to take-off. Ascending and descending jumps have been described in jumping spiders (*Phidippus regius* and *P. princeps*), but outcomes remain unclear, as only one individual was studied (Hill, 2006; Nabawy et al., 2018). In pigeons, potential energy and the power required to fly increases with flight angle (Berg and Biewener, 2008). Thus, systematically probing jump kinematics to targets at different elevations would provide valuable insights.

### Conclusions

Salticids vary greatly in body mass and inhabit a diverse range of microhabitats. After controlling for body mass, we found that nearly all kinematic variables, except jump power, were lower in males compared with females. Jump kinematics have been studied in only a few spider species, with the smallest being males of *M. splendens*. Spiders rely on a combination of hydraulic pressure and muscular action to propel their jump. The jump performance of peacock spiders and other jumping spiders suggests that their jump strategy is more akin to a muscle-actuated system, which operates at several magnitudes slower than spring-actuated systems. A comparative approach to analyse both the jump kinematics and hydraulic mechanism driving the jump is very much needed. Furthermore, analysing the geometry and anatomy of the legs would be fascinating, as they play a crucial role in shaping the fluid dynamics of leg extension.

## Supplementary Material

10.1242/jexbio.249416_sup1 Supplementary information
