# Supplementary material for: Sexual dimorphism in jump kinematics and choreography in peacock spiders
Source: J Exp Biol. 2025 Feb 12;228(3):JEB249416. doi: 10.1242/jeb.249416 (PMC11883279; doi:10.1242/jeb.249416)
Supplement: Supplementary information [file jexbio-228-249416-s1.pdf]

Table S1. The Akaike Information Criteria (AIC) comparing fits between two models. Model 1: variable ~ body size + sex + body size:sex + (1|spider id) and Model 2: variable ~ body size + sex + (1|spider id). Model 2 lacked the interaction term.

	Models	df	AIC
Take-off Velocity	Model 1	6	-106.87
	Model 2	5	-108.147
Acceleration	Model 1	6	-65.064
	Model 2	5	-65.03
Kinetic Energy	Model 1	6	-22.263
	Model 2	5	-22.197
Jump Force	Model 1	6	-65.064
	Model 2	5	-65.029
Jump Power	Model 1	6	58.947
	Model 2	5	57.603
Take-off Duration	Model 1	6	29.165
	Model 2	5	27.295
g-force	Model 1	6	-65.064
	Model 2	5	-65.03

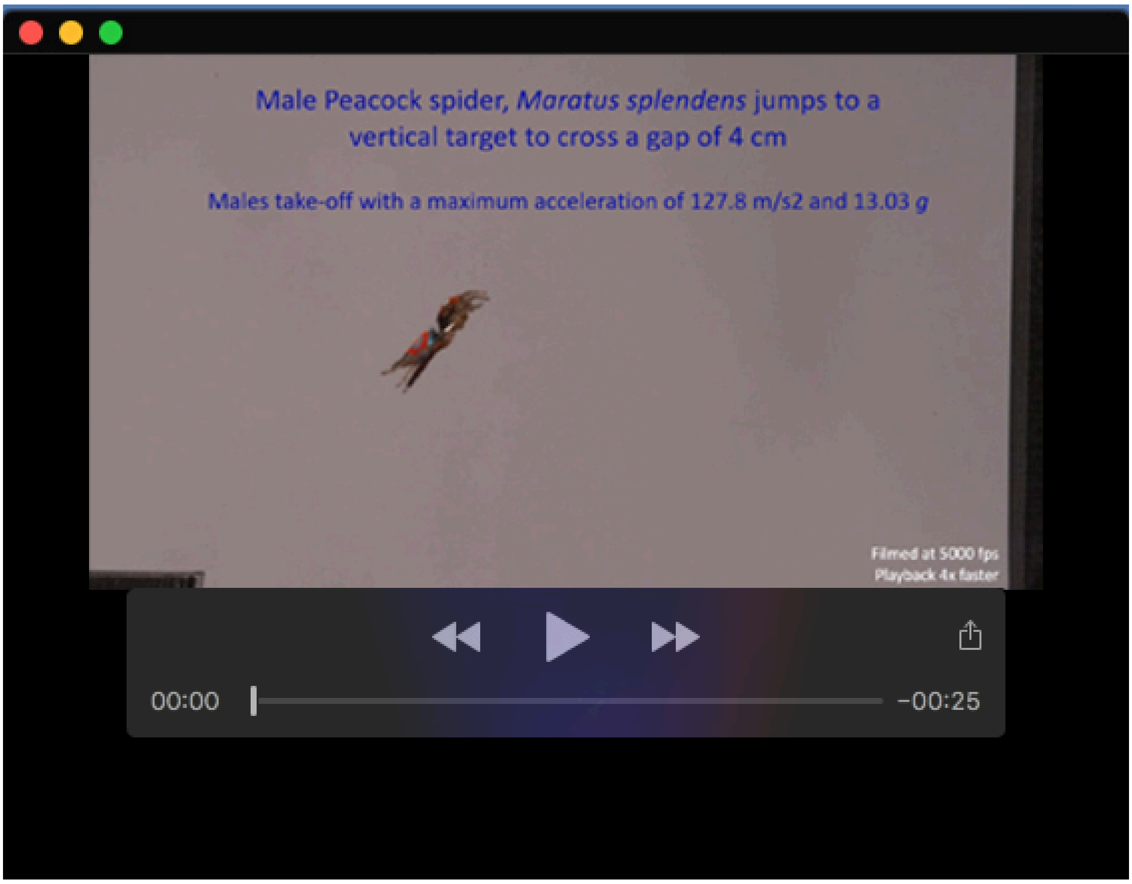

Movie 1. Profile videos of jump of the male and female Australian Splendid Peacock spider, *Maratus splendens*.

Journal of Experimental Biology • Supplementary information
